# Arx Polyalanine Expansion in Mice Leads to Reduced Pancreatic α-Cell Specification and Increased α-Cell Death

**DOI:** 10.1371/journal.pone.0078741

**Published:** 2013-11-13

**Authors:** Crystal L. Wilcox, Natalie A. Terry, Catherine Lee May

**Affiliations:** 1 Department of Pathology and Laboratory Medicine, Children's Hospital of Philadelphia, Philadelphia, Pennsylvania, United States of America; 2 Department of Pediatrics, Division of Gastroenterology, Children's Hospital of Philadelphia, Philadelphia, Pennsylvania, United States of America; 3 Department of Pathology and Laboratory Medicine, University of Pennsylvania School of Medicine, Philadelphia, Pennsylvania, United States of America; Klinikum rechts der Isar der TU München, Germany

## Abstract

ARX/Arx is a homeodomain-containing transcription factor necessary for the specification and early maintenance of pancreatic endocrine α-cells. Many transcription factors important to pancreas development, including *ARX/Arx*, are also crucial for proper brain development. Although null mutations of *ARX* in human patients result in the severe neurologic syndrome XLAG (X-linked lissencephaly associated with abnormal genitalia), the most common mutation is the expansion of the first polyalanine tract of *ARX*, which results primarily in the clinical syndrome ISSX (infantile spasms). Mouse models of XLAG, ISSX and other human *ARX* mutations demonstrate a direct genotype-phenotype correlation in ARX-related neurologic disorders. Furthermore, mouse models utilizing a polyalanine tract expansion mutation have illustrated critical developmental differences between null mutations and expansion mutations in the brain, revealing context-specific defects. Although *Arx* is known to be required for the specification and early maintenance of pancreatic glucagon-producing α-cells, the consequences of the *Arx* polyalanine expansion on pancreas development remain unknown. Here we report that mice with an expansion mutation in the first polyalanine tract of *Arx* exhibit impaired α-cell specification and maintenance, with gradual α-cell loss due to apoptosis. This is in contrast to the re-specification of α-cells into β- and δ-cells that occurs in mice null for *Arx*. Overall, our analysis of an Arx polyalanine expansion mutation on pancreatic development suggests that impaired α-cell function might also occur in ISSX patients.

## Introduction

*Aristaless-related homeobox gene* (*Arx*) encodes a homeodomain containing transcription factor that is expressed in the brain, testis, muscle, pancreas, and digestive tract [1], [2], [3]. In the brain, *Arx* is essential for the proper development and migration of GABA-ergic interneurons and has a role in cortical ventricular zone proliferation [4], [5]. In humans, mutations of *ARX* result in a spectrum of neurologic disorders, the most severe clinical presentation being X-linked lissencephaly associated with abnormal genitalia (XLAG) [5]. XLAG, which has been linked to null and missense mutations in *ARX*, is characterized by a severe brain malformation, termed lissencephaly, corpus callosum agenesis, neonatal-onset intractable epilepsy, and early death [6]. *Arx* null mice phenocopy the clinical presentation of XLAG patients, displaying cortical brain malformations and agenesis with lethality within 24-hours of birth [4], [5]. Histological and molecular analyses reveal a dual function for *Arx* in radial and tangential migration of GABA-ergic interneurons in mice [7].

Interestingly, polyalanine expansion mutations are the most common *ARX* mutations found in humans [8]. ARX contains four polyalanine repeat tracts spaced throughout the open reading frame [9]. In human disease the first two polyalanine repeats that are most often expanded [10]. Patients with these expansion mutations present with severe neurologic phenotypes, including seizures and mental retardation, but without brain malformations [11]. Expansion of the first polyalanine tract by an additional seven alanines has been associated with West Syndrome or infantile spasms (ISSX) [12].

Analyses using genetically modified mouse models have been performed to explore the impact different *ARX* mutations have on neuronal development and cognitive functionality; these models demonstrate a similar genotype-phenotype correlation to humans [13]. Specifically, mouse models with an expansion mutation of the first polyalanine tract of *Arx* reveal that only tangential migration of GABA-ergic interneurons is lost, with no significant impact to radial migration [14], [15]. Thus, it appears that expansion of the first polyalanine tract of *Arx* results in context-specific defects in neural development. In addition to the profound effects *ARX/Arx* mutations have on the brain, they also severely impact the development of other organs. Of note, Itoh and colleagues recently described complete loss of glucagon-producing α-cells in the pancreas of an *ARX*-null XLAG patient [16].

The mammalian pancreas contains an endocrine and exocrine compartment that functions to produce and secrete hormones and enzymes necessary for glucose homeostasis and digestion, respectively [17]. The endocrine compartment is organized into Islets of Langerhans with a core of insulin-producing β-cells and a surrounding mantle of α-, δ-, ε-, and PP-cells producing the hormones glucagon, somatostatin, ghrelin, and pancreatic polypeptide, respectively [18]. *Arx* is expressed in Ngn3^+^ endocrine progenitors during fetal development and later restricted to the α-cell lineage where it is expressed throughout the life of the animal [2]. Loss of glucagon-producing α-cells in XLAG patients suggests that *ARX* is necessary for specification and/or maintenance of this endocrine cell population [16]. Similar observations in the pancreas were also reported in *Arx* null mice in which a complete loss of α-cells was detected [2], [19]. Without Arx function, α-cells are lost while β- and δ-cells simultaneously increase to maintain total endocrine mass [2]. Recently, lineage tracing of these *Arx* ablated α-cells has demonstrated that removal of *Arx* in glucagon^+^ cells results in lineage conversion into an insulin^+^ β-like fate via a bihormonal intermediate [20]. Interestingly, this conversion of α-cells into non-α-cell fates was only seen with loss of *Arx* during the neonatal period, not in adulthood [20].

Previous work has suggested a dual role for Arx in both specification of α-cells and repression of β- and δ-cell fate. However, no studies have investigated the effects of the more common polyalanine expansion mutation on endocrine pancreas α-cell specification and maintenance. Here we show that pancreatic defects associated with this *Arx* expanded mouse model (ArxE) are also context specific. Our results demonstrate a reduced number of glucagon-expressing α-cells in ArxE pancreata, suggesting impaired α-cell specification. However, a subset of α-cells is specified in ArxE mice and these cells do not express other hormones or β-cell specific transcription factors, indicating correct fate determination. Conversely, maintenance of this subset of α-cells is impaired, and these cells are gradually lost through apoptosis over time. Furthermore, unlike *Arx* null mutations, no change in β- or δ- cell mass is observed, suggesting that an expanded Arx protein is still capable of blocking other, non-α-cell fates.

These results describe a unique pancreatic phenotype associated with an *Arx* polyalanine expansion mutation and further illustrate the genotype-phenotype correlation associated with different forms of *ARX/Arx* mutations. Taken together, these findings help elucidate our understanding of Arx-related syndromes outside of the brain as well as characterizing the different roles of *Arx* in α-cell specification versus maintenance.

## Materials and Methods

### Ethics Statement

The Children's Hospital of Philadelphia's Institutional Animal Care and Use Committee (IACUC) approved all animal experiments under the protocol number 2011-10-756. CLM monitored all animal studies.

### Animals and Breeding Strategy

The derivation of mice with an expansion mutation in the first polyalanine tract of Arx has been described previously [15]. Since Arx is located on the X chromosome Arx^Expanded (E)^/_+_ females were bred to Arx^+^/_Y_ males to generate Arx^E^/_Y_ mutant males and Arx^E^/_+_, Arx^+^/_+_, and Arx^+^/_Y_ control females and males, respectively. All male and female control mice were physiologically indistinguishable in all aspects examined and as such were used interchangeably. Mice were maintained on a C57BL/6 background. The Children's Hospital of Philadelphia's Institutional Animal Care and Use committee approved all experiments.

### Immunohistochemistry and Immunoflouresence

All dissections were performed in cold 1× PBS. The entire pancreatic tissue was removed, weighed, and submerged in cold 4% PFA/PBS overnight. Tissues were rinsed in PBS, dehydrated, embedded in paraffin, and sectioned at 8 µm. Antigen retrieval was performed in 10 mmol citric acid buffer (pH 6.0) followed by blocking of endogenous peroxidase and avidin/biotin activity with 3% H_2_O_2_ (Sigma) and avidin/biotin blocking kit (Vector), respectively. Slides were incubated in primary antibody overnight at 4°C. Primary antibodies used were: anti-glucagon (GP 1∶3000, Millipore and Rb 1∶1000, Chemicon), anti-insulin (MS 1∶400, Thermo Scientific and GP 1∶1000, Abcam), anti-PP (Rb 1∶200, Invitrogen), anti-Sst (Rb 1∶200, Invitrogen), anti-ghrelin (Gt 1∶200, Santa Cruz), anti-Pdx1 (Gt 1∶200, Santa Cruz), anti-MafA (Rb 1∶1000, Bethyl), anti-Glut2 (Rb 1∶1000, Millipore), and anti-chromogranin A (Rb 1∶3000, DiaSornin). Appropriate secondary antibodies were added at room temperature (Vector Laboratories). For immunofluorescence, secondary antibodies were conjugated to either Cy2 or Cy3 (Jackson Laboratories) while immunohistochemical detection was obtained with the VECTASTAIN ABC kit and diaminobenzidine tetrahydrocholoride (DAB) substrate (Vector Laboratories). All images utilized in this study were obtained using a Leica DM6000B microscope.

### Real-Time Reverse Transcription PCR Analysis

For expression analysis, whole pancreatic tissue was dissected in cold 1× PBS and homogenized in 1 mL TRIZOL reagent (Invitrogen). RNA was isolated using provided protocol and cDNA synthesized using Oligo-dT, Superscript, and additional necessary reagents (Invitrogen). All quantitative RT-PCR (qRT-PCR) analysis was conducted in duplicate for each specimen including at least three biologic replicates for control and mutant analyzed with reference dye normalization. qRT-PCR analysis was performed using Brilliant SYBR Green PCR Master Mix (Agilent) in a Stratagene Mx3005P Real-time PCR machine. Changes in expression level were determined by calculating and graphing fold change relative to control. Primer sequences are available upon request.

### Hormone Cell Quantification

Total endocrine and hormone cell mass was calculated through the use of Aperio Software (aperio ePathology Solutions, Vista, CA, USA). Two sections per animal were used with four or five animals per group (both control and ArxE) analyzed. Sections were stained for either hormone mass (glucagon, insulin, somatostatin, PP, and ghrelin) or total endocrine mass (ChrgA) using immunohistochemistry. After dehydration and mounting, slides were scanned into the Aperio software and positive hormone area and total pancreatic area determined. Hormone mass and total endocrine mass was than calculated using pancreatic weight. All ArxE mice were males while control specimens consisted of male and female mice (see animal breeding section above). No difference in hormone or total endocrine mass was seen between any of the controls utilized.

### Statistical Analysis

Error bars display standard error of the mean (SEM). Significance was determined using a two-tailed Student's t-test and results considered significant when p≤0.05.

## Results

### ArxE mice retain a subset of glucagon-producing α-cells at embryonic day (E) 15.5

To determine the effect of an *Arx* expansion mutation on α-cell specification and maintenance, hemizygous Arx^Expanded^/_Y_ (referred to as ArxE from here on) mutant mice were obtained by crossing heterozygous Arx^Expanded^/_+_ females to wild-type Arx^+^/_Y_ males. ArxE mice were born in the expected Mendelian ratios with birth weights similar to their littermate controls. However, ArxE mice displayed slower postnatal growth and by postnatal day 6 (P6) were significantly smaller than control littermates (Terry and May, unpublished data). We did not observe any significant changes in pancreatic weight, morphology, or histology between ArxE mice and controls at any time point (data not shown).

**Figure 1:**
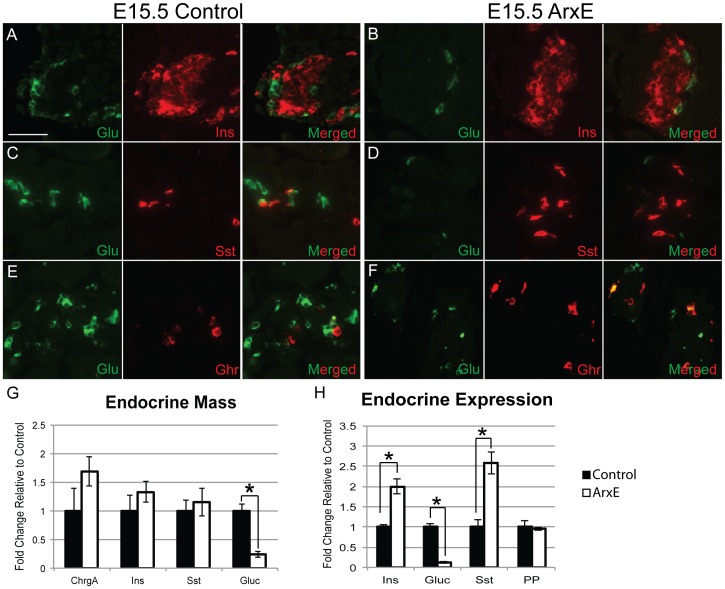
ArxE mice are able to specify a subset of α-cells at E15.5. (**A–F**): Control and ArxE E15.5 pancreatic sections were stained for glucagon (green) and insulin (red; A–B), somatostatin (Sst; red; C–D), and ghrelin (Ghr; red; E–F). Scale bar denotes 50 µm. (**G**): Quantification of total endocrine (ChrgA) and β-, δ-, and α-cell mass in control (black bar) and ArxE (white bar) pancreata. (**H**): Quantification of transcript levels for each endocrine hormone in control (black bar) and ArxE pancreata (white bar) at E15.5 using qRT-PCR. All results are graphed as fold change relative to littermate controls ± standard error of the mean. Significance is denoted with (*) when p≤0.05. All analysis consists of 4–5 animals per group.

To explore a possible pancreatic defect, immunostaining was performed in control and ArxE pancreata at E15.5 to examine the presence, localization, and segregation of each endocrine hormone. Immunostaining for glucagon demonstrated a dramatic reduction in the number of glucagon^+^ α-cells at E15.5 in ArxE mice (Fig. 1A–F). However, unlike in *Arx* null animals with their complete loss of α-cells, a subset of α-cells was specified in ArxE mice [2], [19]. Furthermore, these remaining glucagon^+^ cells did not co-express insulin or somatostatin, suggesting proper α-cell fate determination (Fig. 1A–D). Co-staining for glucagon and ghrelin did reveal 30% co-localization in both control and ArxE pancreata, similar to what has been previously described in wild-type pancreata (Fig. 1E–F) [21]. PP-cells are not normally present at E15.5 and immunostaining for glucagon and PP did not reveal any precocious specification of this cell type in ArxE mice (data not shown) [17]. Consistent with our qualitative analysis, morphometric studies showed that while α-cell mass was significantly decreased to 20% of wild-type levels, this was not accompanied by a change in β- or δ-cell mass (Fig. 1G). Total endocrine mass was not significantly altered at this time point.

Although the endocrine mass of β- and δ-cells was not changed, we also measured the expression levels of the hormone genes in ArxE mice using quantitative reverse transcription PCR (qRT-PCR). The expression level of glucagon was significantly reduced in ArxE pancreata, as expected (Fig. 1H). However, both insulin and somatostatin transcript levels were significantly upregulated in ArxE pancreata when compared to littermate controls (Fig. 1H). No significant difference in the expression level of PP was observed in ArxE mice, further indicating that there was no precocious expression of PP upon ArxE mutation (Fig. 1H). Since β- or δ-cell mass was not increased, our qRT-PCR results suggest that there is a significant upregulation of hormone gene expression per endocrine cell. These data demonstrate that the majority of α-cell specification is impaired in ArxE mice but, for a subset of α-cells that are specified, fate determination remains intact without any co-expression of other endocrine hormones.

### Postnatal loss of the α-cell lineage in ArxE mice

**Figure 2:**
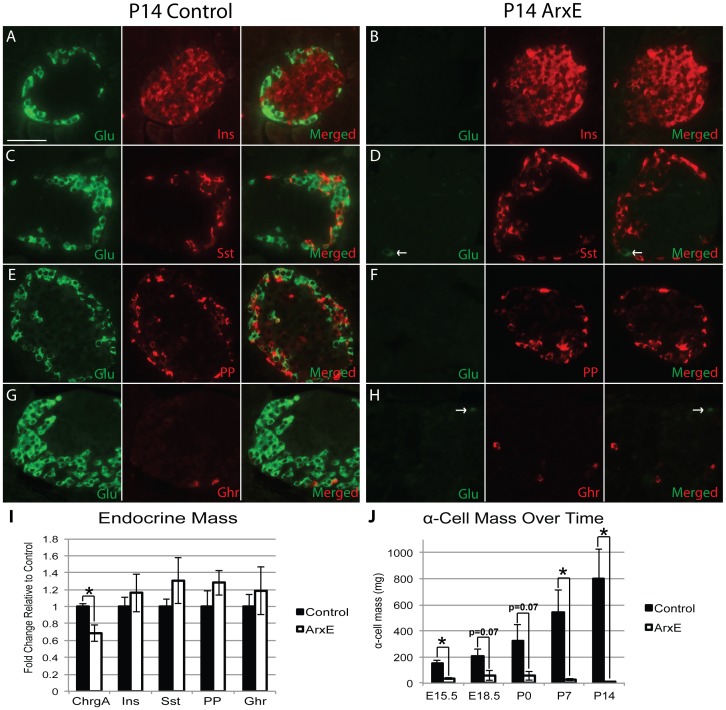
ArxE mice have almost complete loss of α-cell fate by P14 with a concomitant decrease in total endocrine mass, but no change in β- and δ-cell mass. (**A–H**): P14 pancreatic sections were stained for glucagon (green) and insulin (red; A–B), somatostatin (Sst; red; C–D), PP (red; E–F), and ghrelin (Ghr; red; G–H). Scale bar denotes 50 µm. (**I**): Quantification of endocrine hormone mass including total endocrine mass (ChrgA), insulin, somatostatin, PP, and ghrelin displayed as fold change in ArxE mice (white bar) relative to control (black bar). (**J**): Analysis of glucagon mass over time starting at E15.5 and ending at P14 in control (black bar) and ArxE (white bar) pancreata. Resulting p value is listed. (*) denotes significance where p<0.05. Error bars represent standard error of the mean (I, J). For all analysis 4–5 animals per group were analyzed with all ArxE mice being males and control mice consisting of male and female mice.

To determine the fate of this remaining subset of α-cells in ArxE mice, we analyzed control and ArxE mutant pancreata two weeks after birth (P14). Morphometric analysis showed that α-cell mass was reduced by 99.5% with only scattered glucagon^+^ cells remaining (Fig. 2A–H, J). Immunostaining and quantification of β-, δ-, PP-, and ε-cell mass at P14 did not reveal any significant differences in quantity or localization of these endocrine cell types in ArxE mice (Fig. 2A–I). Finally, we observed a significant 30% reduction in total endocrine mass in P14 ArxE mice as measured by chromogranin A immunolocalization (Fig. 2I). This reduction in total endocrine mass can be attributed to the near total loss of α-cells in the ArxE mice at this age. These data suggest that α-cells are eliminated in ArxE mutant mice and not reallocated to a β- or δ-cell fate.

To examine when the loss of the α-cell lineage occurs, we measured α-cell mass at multiple time-points, starting at E15.5 (Fig. 2J). As reported above, a subset of α-cells is properly specified in E15.5 ArxE pancreata; however, these cells are gradually lost over time. From our morphometric analyses of E15.5, E18.5, P0 and P14 pancreata, we conclude that this α-cell population is lost gradually and without reallocation to a β- or δ-cell fate, resulting in a significant reduction in total endocrine cell mass postnatally.

### ArxE α-cells do not express β-cell specific transcription factors

**Figure 3:**
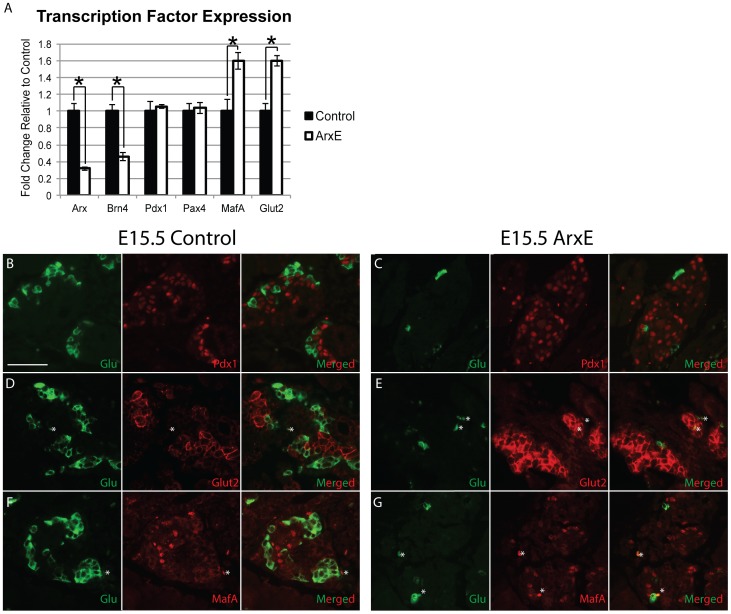
Specified α-cells in ArxE mice do not misexpress β-cell specific transcription factors at E15.5. (**A**): Quantification of transcript levels for α- and β-cell specific transcription factors graphed as fold change in ArxE pancreata (white bar) relative to littermate control (black bar). Error bars represent standard error of the mean. Significance when p≤0.05, is denoted with (*). (**B–G**): Control and ArxE E15.5 pancreatic sections were stained for glucagon (green) and Pdx1 (red; B–C), Glut2 (red; D–E), and MafA (red; F–G). Five specimens were analyzed for each group and representative images taken. (*) denotes autofluorescence of red blood cells. Scale bar denotes 50 µm.

To further examine whether α-cell specification is properly executed in ArxE pancreata, we employed qRT-PCR to analyze the expression of α- and β-cell specific transcription factors in ArxE and control pancreata at E15.5. The α-cell specific transcription factors Arx and Brn4 were significantly downregulated in ArxE mice to approximately 30% of wild-type levels (Fig. 3A). This downregulation is similar to the reduction in α-cell mass and thus simply reflects the reduced proportion of α-cells in the ArxE mice. Strikingly, examination of β-cell specific factors MafA, Glut2, Pdx1, and Pax4 demonstrated a significant upregulation of MafA and Glut2 messenger RNA (mRNA) levels, while Pdx1 and Pax4 expression was not altered (Fig. 3A).

To determine if the significant upregulation of MafA and Glut2 mRNA levels in ArxE mice results in misexpression of these factors in α-cells, we performed immunostaining for glucagon, Pdx1, Glut2, and MafA (Fig. 3B–G). Similar to control animals, no colocalization of glucagon with Pdx1, Glut2, or MafA was observed in ArxE pancreata at E15.5 (Fig. 3B–G). These data indicate that while an *Arx* expansion mutation results in upregulation of MafA and Glut2 transcript levels, this change does not lead to misexpression of the protein in ArxE E15.5 glucagon^+^ α-cells. When combined with previous results, these data indicate that α-cell fate determination has occurred normally in the remaining glucagon^+^ cells.

### ArxE pancreata contain more apoptotic glucagon^+^ cells

**Figure 4:**
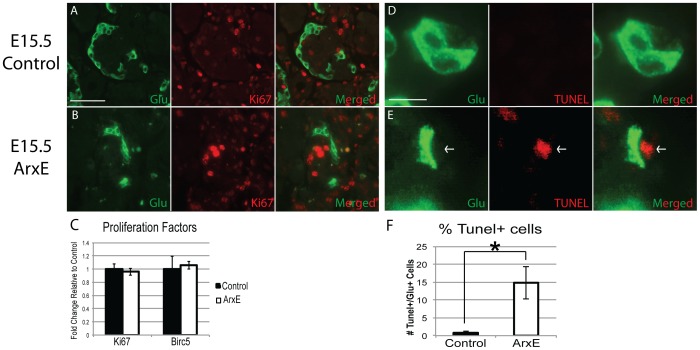
There is a drastic increase in the percentage of apoptotic glucagon^+^ cells in ArxE mice, but no apparent change in proliferation. (**A–B**): Control and ArxE E15.5 pancreatic sections were stained for glucagon (green) and Ki67 (red). Scale bar denotes 50 µm. (**C**): qRT-PCR analysis for two markers of proliferation, Ki67 and Birc5, in E15.5 control (black bar) and ArxE pancreata (white bar). Results are graphed as fold change relative to littermate control ± the standard error of the mean. (**D–E**): Control and ArxE E15.5 pancreatic sections were stained for glucagon (green) and TUNEL (red). Arrow points to TUNEL^+^ cell. Scale bar denotes 10 µm. (**F**): Quantification of the percentage TUNEL^+^glucagon^+^ cells over total counted glucagon^+^ cells in control and ArxE E15.5 pancreatic sections. All glucagon^+^ cells in five different pancreatic sections from both control (black bar) and ArxE (white bar) mice were counted and determined to be TUNEL positive or negative. The percentage of TUNEL^+^glucagon^+^ cells was calculated and graphed as ± standard error of the mean. (*) denotes significance where p≤0.05. Between 4 and 5 animals were examined per group for each analysis.

There are two possible causes for the apparent loss of α-cells in ArxE pancreata: reduced proliferation or increased apoptosis. Proliferation was measured by examining the localization and quantity of proliferating glucagon^+^ α-cells using the proliferation marker Ki67. No change in the number of proliferating, Ki67^+^ α-cells was noted in ArxE mice when compared to control littermates at E15.5 (Fig. 4A–B). Furthermore, expression analysis using qRT-PCR for Ki67 and Birc5 (another proliferation marker) did not reveal any differences between control and ArxE pancreata (Fig. 4C). These data demonstrates that α-cells proliferate normally in ArxE mice at E15.5.

To explore whether changes in the rate of apoptosis may have contributed to α-cell loss we performed TUNEL assays. TUNEL assay and glucagon immunostaining demonstrated an increase in the number of TUNEL^+^/glucagon^+^ cells in ArxE pancreata at E15.5 (Fig. 4D–E). Quantification of the percentage of glucagon^+^ that were TUNEL^+^ in control and ArxE pancreata demonstrated a significant and profound increase in the percent apoptotic α-cells in ArxE pancreata (Fig. 4F). Taken together, these results demonstrate that an increase in apoptosis is the major contributor to the temporal α-cell loss seen in ArxE pancreata.

## Discussion

**Figure 5:**
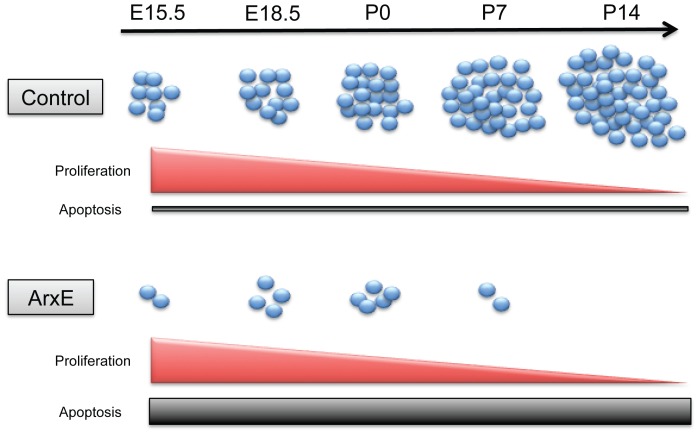
ArxE mice are able to correctly specify a subset of α-cells, but α-cells are gradually lost through apoptosis. Model demonstrating normal proliferation, but increased apoptosis in ArxE mice. Normal proliferation during embryonic time points maintains the α-cell lineage by replacing cells lost to apoptosis. However, proliferation slows during the neonatal stage leading to loss of the α-cell lineage.

This study demonstrates that expansion of the first polyalanine tract of *Arx* results in impaired specification and maintenance of endocrine α-cells through a mechanism of programmed cell death, as opposed to α-cell fate re-specification. We show that only a subset (20%) of α-cells is present in E15.5 ArxE pancreata. Even these glucagon^+^ α-cells are not maintained over time and eventually undergo apoptosis, leading to a complete absence of the α-cell lineage and a significant decrease in total endocrine cell mass by P14. While cell proliferation is a major mechanism to expanding α-cell mass during development, we did not detect a significant change in the rate of proliferation in ArxE pancreata. Analysis of apoptosis by TUNEL suggests that programmed cell death is the principal cause of α-cell loss after their initial specification (Fig. 5).

### ArxE mice reveal context specific defects in α-cells that differ from Arx null mice

Our study reveals a novel and unique impact of ArxE mutations in the pancreas. Phenotypes found in ArxE mice differ from those reported in the *Arx* null mouse models in the following ways: (1) there is no change in β- and δ-cell mass, (2) there is a significant reduction in total endocrine cell mass, and (3) a small number of α-cells are present during embryogenesis [2]. *Arx* null mutations result in loss of α-cell specification in which glucagon^+^ cells are never present, even at embryonic time points [2]. Loss of *Arx* in these endocrine progenitors results in reallocation of these cells into a β- or δ-cell fate, thus maintaining total endocrine mass [2], [20]. Conversely, ArxE mice are able to specify a small subset of α-cells and, more importantly, repress non-α-cell fates, including β and δ. This retained repressive ability results in normal β- and δ-cell mass even with the eventual complete loss of the α-cell lineage through apoptosis.

### ArxE mice display selective derepression similar to previous neuronal studies

Cell culture based mechanistic studies have demonstrated that Arx associates with the groucho-family corepressor Tle1 [22]. This association is carried out through Arx's octapeptide domain and results in increased repressive activity [22]. Examination of this association in an expanded neuronal model demonstrates a decreased, but not complete loss, of Arx-Tle1 protein binding [23]. Furthermore, neuronal studies have demonstrated that expansion of the first polyalanine tract of Arx results in selective derepression of a subset of Arx targets [23]. It is hypothesized that loss of association with specific co-repressors (if not Tle1 itself) results in this selective derepression [23]. Interestingly, we show here that ArxE mice do not misexpress either the endocrine hormones insulin and somatostatin or β-cell specific transcription factors in embryonic α-cells. Thus, it appears that at least part of Arx's repressive abilities are intact. While no misexpression of β-cell specific factors was observed via immunoflouresence in ArxE α-cells, expression levels of MafA, Glut2, and insulin were significantly upregulated by qPCR. There are two possible explanations for these findings. First, MafA Glut2, and insulin could be upregulated in ArxE α-cells; however, this upregulation only results in a low level of protein, which is not detectable by immunostaining. Alternatively, the resulting loss of α-cells in the ArxE mutant mice could indirectly lead to upregulation of MafA, Glut2, and insulin within β-cells. Based on the data presented in this study, we believe that the observed upregulation of these three genes at the transcript levels is an indirect effect in β-cells. Future studies using chromatin immunoprecipitation to determine direct targets of Arx in the pancreas will serve to clarify these findings.

### Comparing ArxE and Arx null mouse models demonstrate a direct genotype-phenotype correlation

In the nervous system, there appears to be a direct genotype-phenotype correlation associated with various *Arx* mutations [24]. Our study demonstrates that this correlation is likely to be applicable in the pancreas as well. In the brain, more severe phenotypes are attributed to null and missense mutations of *Arx* [8]. In the pancreas, the *Arx* null mutation has an earlier onset with complete loss of glucagon-expressing α-cells [2]. Although the α-cell population is almost completely lost by P14 in the ArxE mice, their survival curve compared to the global null mice is improved, and a fraction of α-cells are initially specified. At present, we cannot determine if the improved survival is caused by the maintenance of a fraction of α-cells at birth, or the other tissues in which ArxE is expressed. Although it has been suggested that hypoglycemia is the cause of early fatality, blood sugar levels in the pancreas are likely also affected by the diarrhea in the ArxE mouse model (Terry and May, unpublished data).

## Conclusion

In conclusion, this study demonstrates dual functions for Arx in α-cell gene activation and β-cell gene repression during fate specification and maintenance. Utilizing a mouse model with an expansion mutation of the first polyalanine tract of *Arx*, we demonstrate impaired, but not eliminated α-cell fate specification. Although α-cell number is reduced, proper fate determination is observed in the remaining α-cells. However, these cells eventually undergo apoptosis, which leads to complete loss of α-cell fate postnatally. β- or δ-cell mass is not increased, and there is a significant decrease in total endocrine cell mass, attributed to α-cell death. Our study begins to explore the more common polyalanine expansion, non-null *Arx* mutations and the effect they have on α-cell specification and maintenance. Being able to separate the dual function for gene activation and gene repression leading to fate specification and maintenance will lead to a better understanding of the clinical presentation of ARX-related disorders and help in designing future therapeutic treatments.
